# Technical note: Vendor‐agnostic water phantom for 3D dosimetry of complex fields in particle therapy

**DOI:** 10.1002/acm2.12996

**Published:** 2020-09-29

**Authors:** Christoph Schuy, Yuri Simeonov, Marco Durante, Klemens Zink, Uli Weber

**Affiliations:** ^1^ GSI Helmholtzzentrum für Schwerionenforschung Darmstadt Germany; ^2^ Physics Department Technische Universität Darmstadt Darmstadt Germany; ^3^ Institute of Medical Physics and Radiation Protection (IMPS) THM University of Applied Sciences Giessen Giessen Germany

**Keywords:** 2D ionization chamber array detectors, 3D dosimetry, particle therapy, water phantom

## Abstract

**Purpose:**

Three‐dimensional (3D) dosimetry is a necessity to validate patient‐specific treatment plans in particle therapy as well as to facilitate the development of novel treatment modalities. Therefore, a vendor‐agnostic water phantom was developed and verified to measure high resolution 3D dose distributions.

**Methods:**

The system was experimentally validated at the Marburger Ionenstrahl‐Therapiezentrum using two ionization chamber array detectors (PTW Octavius 1500XDR and 1000P) with 150.68 MeV proton and 285.35 MeV/u ^12^C beams. The dose distribution of several monoenergetic and complex scanned fields were measured with different step sizes to assess the reproducibility, absolute positioning accuracy, and general performance of the system.

**Results:**

The developed system was successfully validated and used to automatically measure high resolution 3D dose distributions. The reproducibility in depth was better than ±25 micron. The roll and tilt uncertainty of the detector was estimated to be smaller than ±3 mrad.

**Conclusions:**

The presented system performed fully automated, high resolution 3D dosimetry, suitable for the validation of complex radiation fields in particle therapy. The measurement quality is comparable to commercially available systems.

## 1 INTRODUCTION

State‐of‐the‐art particle therapy can deliver highly conformal dose distributions to target volumes while sparing healthy tissues due to their advantageous inverse depth dose distributions and increased biological effectiveness compared to other forms of external radiation therapy.^1^ To leverage the physical and biological advantages of particles for better health outcomes for patients, strict patient‐specific quality assurance (PSQA) is a necessity.^2^ Classic three‐dimensional (3D) dosimetry concepts for PSQA, employing multiple pin‐point ionization chambers,^3^ radiographic films or gels^4^ have either limited spatial resolution (pin‐point ionization chambers) or a complex dependence on linear energy transfer (LET) and particle energy (radiographic films or gels). Medical two‐dimensional ionization chamber arrays^5^ or novel approaches using GEM‐based detectors^6^ offer a comparatively high spatial resolution and immediate data readout, but are often not directly usable within a water phantom and/or are comprised of non‐water‐equivalent materials. Even though the stopping power of materials such as water‐equivalent plastics are comparable to water, nuclear interactions of heavier ions, such as carbon, and scattering of light ions, such as protons, will be different in these materials and will have detrimental effects on the comparability of the measurement outcome.^7^

Additionally, high‐resolution, 3D dosimetry is an absolute necessity for benchmarking and for further optimization of different beam application modalities. Two‐dimensional passive range modulators (2DRM), like 2D SOBP modulators^8^ or ripple filters,^9^ are still under active development and three dimensional passive range modulators (3DRM) are showing great potential for the treatment of moving tumors^10^ and may be crucial for new treatment modalities, such as FLASH irradiations.^11^, ^12^

To the best of our knowledge, only the company IBA (Louvain‐la‐Neuve, Belgium) offers a commercial medical product^13^ suitable for 3D dosimetry in water for particle therapy, based on a two‐dimensional ionization chamber array.^14^ However, this dosimetry system is highly integrated into the IBA ecosystem of accelerators and quality assurance solutions making it difficult to use at non‐IBA medical accelerators, such as the SIEMENS‐built medical ion beam facilities in Marburg (MIT), Shanghai (SPHIC) or Heidelberg (HIT). The applicability of such a highly optimized medical dosimetry system to the development of different beam application modalities, such as 2DRMs and 3DRMs, is hindered further by the inability to extend its capabilities or to optimize the system for a specific experimental measurement task.

Therefore, a universal and vendor‐agnostic water column for 3D dosimetry in particle therapy was developed [**W**at**ER** column for 2D io**N**ization chamb**E**r a**R**ray detectors (WERNER)]. The system is comprised of a PMMA water tank with a stepper‐driven watertight detector holder, a standard medical two‐dimensional ionization chamber array as well as analog and digital input and outputs (AD I/O). The full system is controlled via a LabVIEW application and synchronized to the dose delivery system of the medical accelerator using commonly available digital signals. In this work, we describe the current iteration of WERNER, optimized for the use at MIT and for the PTW 2D ionization chamber arrays OCTAVIUS 1500XDR and 1000P. The system recorded more than 50 000 individual dose points in <5 min per measurement to validate and benchmark complex fields generated by different 2DRMs and 3DRMs.

## 2 MATERIALS AND METHODS

**Fig. 1:**
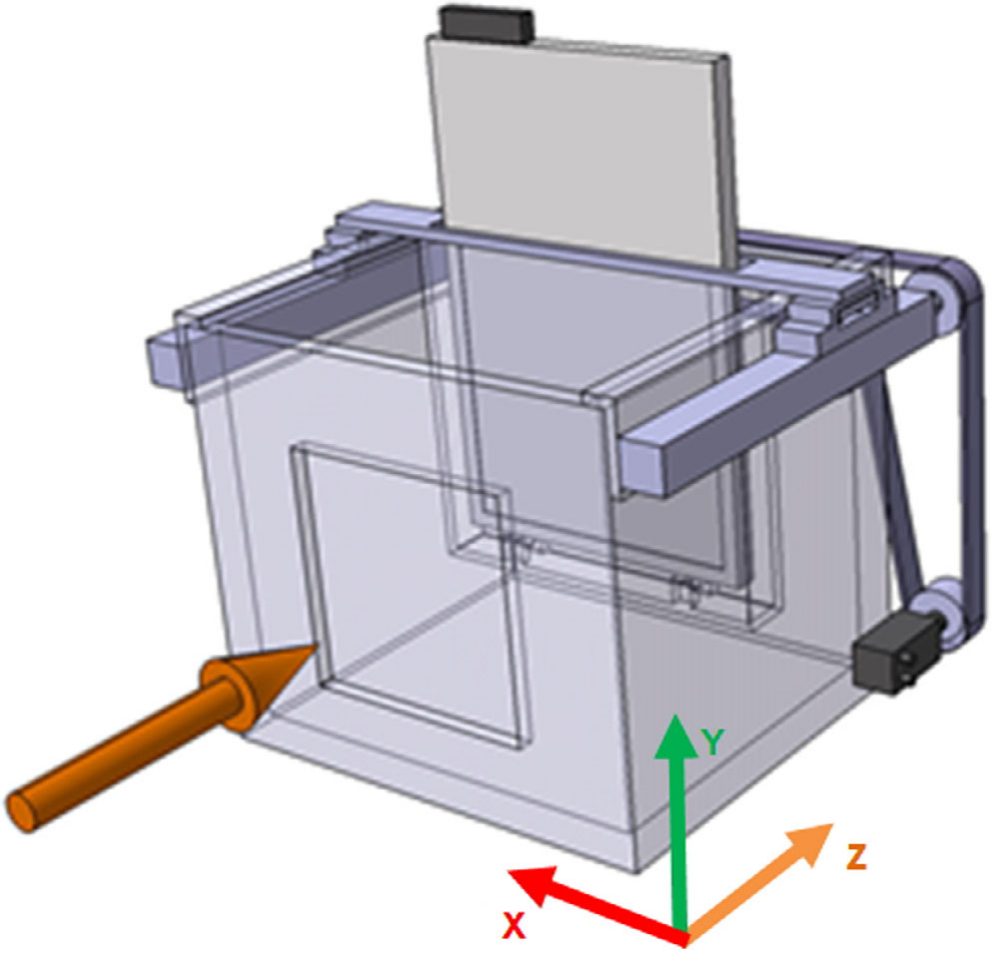
Schematic view of the developed water column. A full three‐dimensional representation can be found in the supplements

The development of the universal water column for 2D ionization chamber array detectors is described in detail in the following paragraphs. A schematic drawing of the full system is shown in Fig. 1.

### 2.A Mechanical setup

The water column consists of a cuboid‐shaped PMMA tank (inner dimensions: 400 × 335 × 350 mm^3^) and 20 mm thick outer walls for mechanical stability. To minimize the nonwater material thickness, a 5 mm thick PMMA beam entrance window (dimensions: 220 × 220 mm^2^) is embedded into the front‐facing wall. Two ball screw actuators (GUNDA ELS‐R25), connected to a single stepper motor (GUNDA SM23Hz) via a drive belt, are mounted to the side of the water column to ensure highly precise and synchronous movement of the detector holder over the full length of the water column. Motor movement is governed by an external stepper motor driver (GUNDA BAC100), which is controlled by a LabVIEW application via RS485. The watertight PMMA detector holder is connected to both actuators via a spring‐loaded connection to prevent excessive forces during fast acceleration and, additionally, is stabilized by a top‐mounted aluminum bracket to prevent oscillation. The detector stands on a raised platform within its holder to offer protection in case of water leakage and to guarantee a reproducible and tight fit to the 5 mm front plate of the holder. To minimize the production of waves in the water tank, the nominal movement speed of the detector was set to 10 mm/s.

### 2.B Array detectors

All tests were conducted with two different PTW ionization chamber array detectors: the OCTAVIUS 1500XDR and the OCTAVIUS 1000P. Both detectors are specified for use in particle therapy and share a common detector housing geometry, readout characteristics and readout software and are, therefore, easily interchangeable during an experiment.

The OCTAVIUS 1500XDR^15^ employs a matrix of 1405 vented cubic ionization chambers, uniformly arranged on a 27 × 27 cm^2^ grid with a diagonal center‐to‐center spacing of 7.1 mm. The active area of each ionization chamber is 4.4 × 4.4 mm^2^. The OCTAVIUS 1000P, a prototype detector, similar to the OCTAVIUS 1000SRS^15^ but optimized for particle therapy, consists of 977 vented ionization chambers with an active area of 2.3 × 2.3 mm^2^ each and spread over an area of 11 × 11 cm^2^. In the inner 5.5 × 5.5 cm^2^ area ionization chamber spacing is 2.5 mm, whereas the spacing outside is 5 mm. Both detectors were used with the ^60^Co calibration, as provided by the manufacturer, and are, therefore, only suitable for relative dosimetry of ^12^C beams. Absolute dose calibration of the detectors is possible using external reference fields, but is not within the scope of this work.

### 2.C Measurement control

The PTW two‐dimensional ionization chamber array is operated with a beta version of its standard software — BeamAdjust (V2.1 T182) — which offers a time resolved (max. 10 Hz) and timestamped detector readout. The movement of the detector, the AD I/O interface (NI USB‐6215) and the synchronization with the dose delivery system are managed via an in‐house developed LabVIEW‐based control software. In order to perform fully automated measurements of complex dose distributions, the user must provide a set of measurement positions to the control software and an appropriate treatment plan to the dose delivery system. The treatment plan is modified by the user in a way that it is automatically repeated once for every depth specified by the user. At the end of each repetition, a digital signal (End‐Of‐Plan) is generated by the dose delivery system, triggering the control software to change the detector position during a spill pause. Additionally, several other digital signals that are generated by the dose delivery system (e.g. Beam‐Gate) are registered by the control software and are used to verify that motor movement is finished before the next plan irradiation starts. All external signals and information on the stepper motor are timestamped and logged in an output file.

### 2.D Data processing

The output files created by the detector and the water column control software are processed offline by an in‐house developed MATLAB program. This software combines the. XCC files generated by BeamAdjust, containing the accumulated dose for all chambers every 100–800 ms (depending on detector setting), with the log file generated by WERNERs control software, containing timestamped information on the position of the detector as well as the state of the accelerator. The information is then used to match all readout frames of the detector to their corresponding detector positions and the respective spills of the synchrotron. The result is a two‐dimensional dose distribution for each measured detector position, which can then be combined to obtain a high resolution, three‐dimensional dose distribution.

## 3 RESULTS

**Fig. 2:**
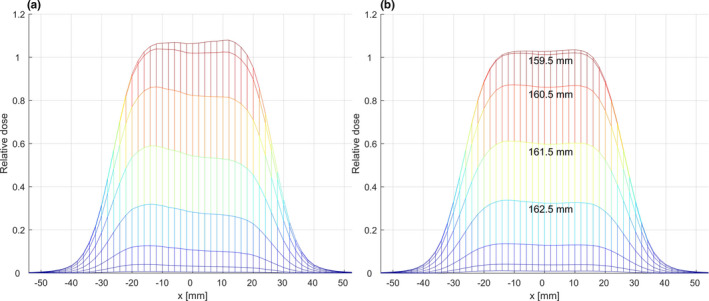
Improper mechanical alignment will result in a tilt of the lateral dose profiles especially in high dose gradient regions (Panel a) compared to a properly calibrated system (Panel b)

The described water column was validated both mechanically, using high precision dial gauges (Garant 43 2210_10/58), and a two‐dimensional inclinometer (DigiPas DWL‐1500XY) as well as experimentally, exploiting the sharp dose gradients used in carbon therapy. The mechanical validation could only be performed relative to the detector holder and is therefore not able to verify the absolute position of the detector within its holder. In general, the mechanical validation was in good agreement with the validation using particle beams and, therefore, is not discussed in detail. However, it is important to note that the same tools and techniques are used for the mechanical calibration after each assembly. The impact of improper mechanical calibration is highlighted in Fig. 2.

Validation of the 3D dosimetry capabilities of the system with ion beams was performed at MIT using 150.68 MeV proton and 285.35 MeV/u ^12^C beams with the PTW 1500XDR and the PTW 1000P. A 50 × 50 mm^2^ rectangular scanned field was measured with two position patterns of varying step size and a detector readout time of 400 ms. Additional comparative measurements with the PTW PeakFinder^4^ were performed with 150.68 MeV protons and a constant step size of 1 mm.

The coarse position pattern, in case of 3D dosimetry, consisted of 60 individual measurement positions with a minimum step size of 1 mm in the Bragg peak region, while the fine position pattern used a minimum step size of 250 µm in the Bragg peak region and 130 individual measurement positions in total. The center of the water tank was positioned at isocenter using the in‐room positioning lasers. Including all nonwater materials of the water tank, the nozzle detectors and air gaps, the minimum measurable water equivalent thickness (WET) with the two PTW detectors was 32.2 and 32.4 mm respectively. The maximum achievable WET was set via a software limit to 280 mm. The absolute error of the WET at any given depth consists of thickness deviations in all nonwater materials of WERNER, penetrated by the beam and the positional accuracy of the movement system. Thickness deviations on the milled 5 mm PMMA entrance window of the water tank and the detector holder are estimated to be ±0.1 mm each. The positional accuracy and short‐term reproducibility of the distance between the water tank window and the detector holder front was measured using high precision dial gauges and was estimated to be ±0.1 mm and ±10 µm respectively.

**Fig. 3:**
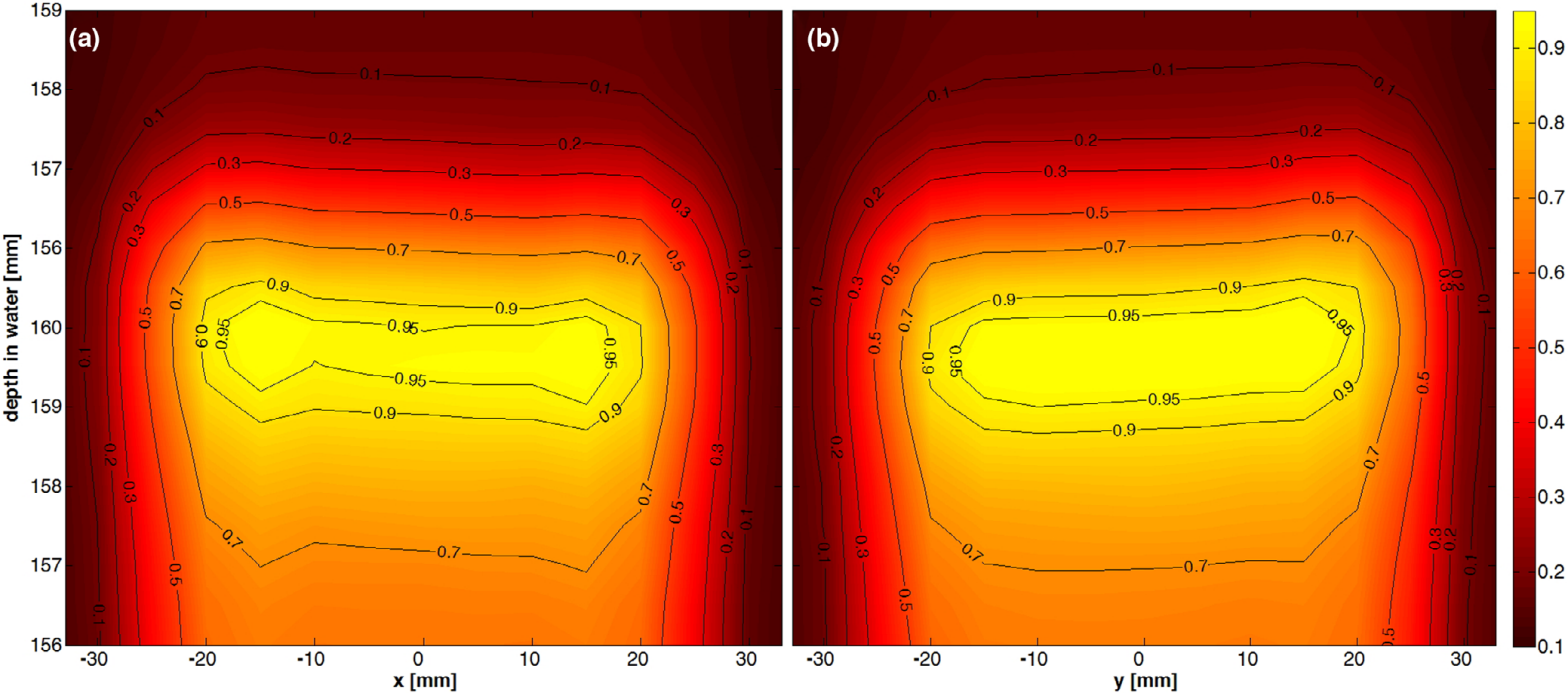
Normalized dose distribution over the x‐z (Panel a), respectively, y‐z plane (Plane b) through the isocenter and zoomed in to highlight the high gradient region around the Bragg peak

Figure 3 shows the zoomed in, normalized dose distribution of a rectangular scanned field in the horizontal (Panel a) and vertical direction (Panel b) vs WET as well as the corresponding isodose lines for 285.35 MeV/u ^12^C, measured with the fine movement pattern and the OCTAVIUS 1500XDR. The agreement in dose of repeated measurements with different step sizes was generally better than 1% at nominally equal positions. The measurement points in the high dose gradient region after the Bragg peak show a spatial reproducibility in depth of around ±25 µm. It is important to note that the 3 mm distal extension of each individual ionization chamber (OCTAVIUS 1500XDR) leads to a slight broadening of the depth dose distribution, especially in high gradient regions. Possible rotations of the detector were estimated by calculating the tilt of the isodose lines in the high dose gradient region after the Bragg peak and are typically ≤±3 mrad in all planes.

**Fig. 4:**
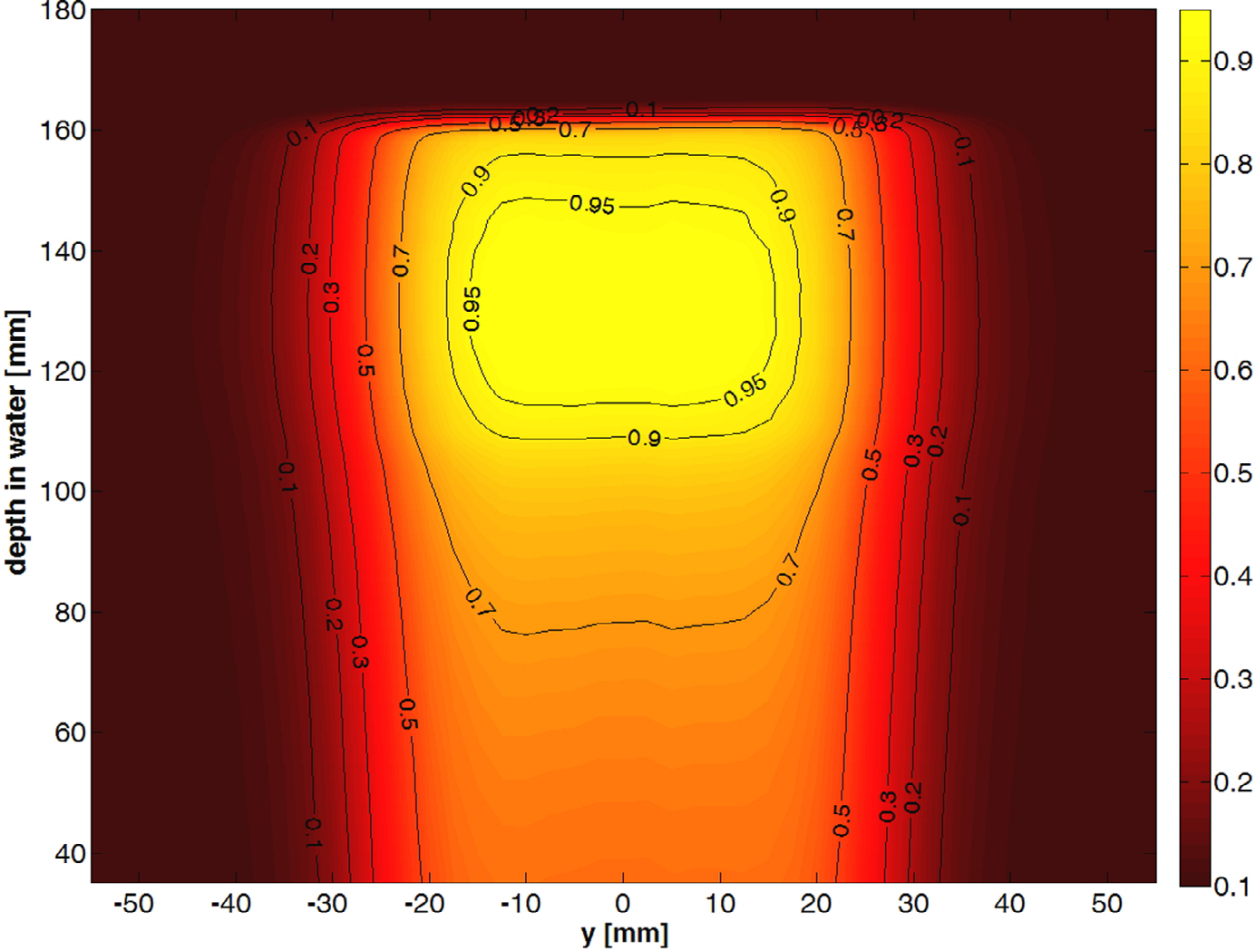
Normalized dose distribution of a cubic SOBP (40 × 40 × 40 mm^3^) for a 150.68 MeV proton beam over the, x‐z plane through the isocenter

A more realistic case of a normalized dose distribution produced by an optimized SOBP modulator and 150.68 MeV protons, measured with the OCTAVIUS 1000P and the coarse movement pattern is shown in Fig. 4. The measurement consists of around 60,000 individual dose points and was measured in <8 min.

**Fig. 5:**
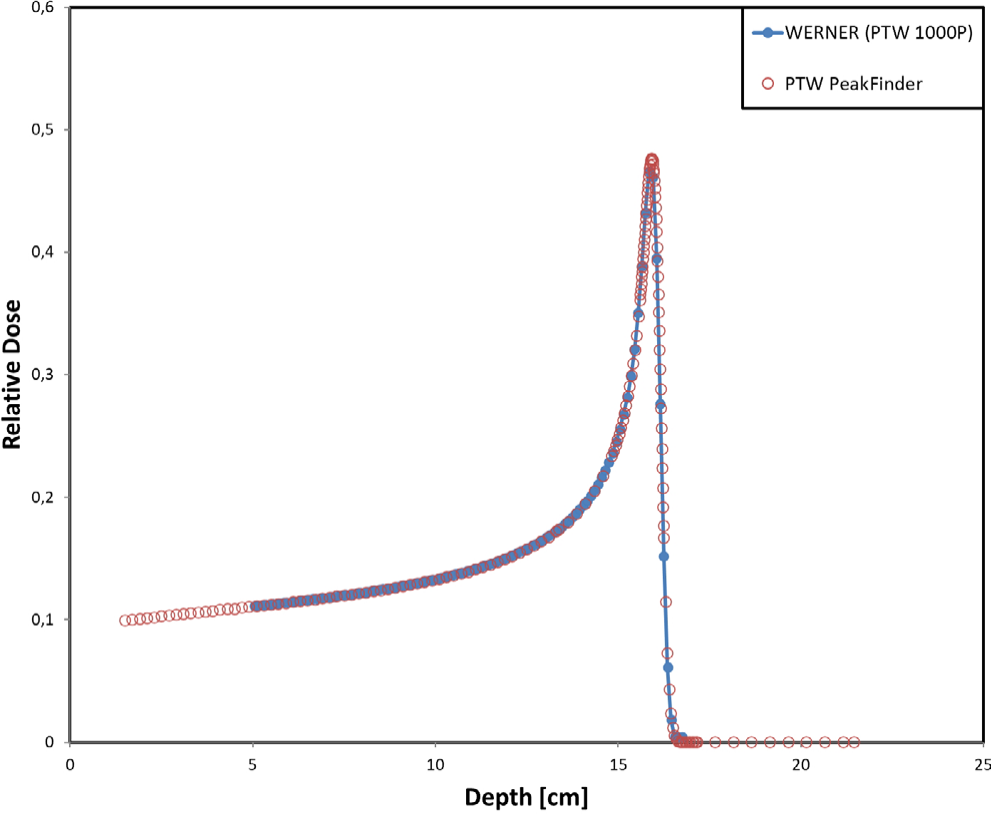
Comparison of the presented system and the PTW PeakFinder for 150.68 MeV protons on water. For easier comparability, the PeakFinder measurement points are shifted − 0.5 mm in depth and scaled by 0.097 in relative dose

In order to compare the performance of WERNER to a commercially available medical system, we performed additional measurements with 150.68 MeV protons and the PTW PeakFinder. The PeakFinder is a water column system, designed for high precision Bragg curve measurements and is routinely used for quality assurance in particle therapy centers. The signal of the individual chambers of the array detectors was integrated per measurement position and the results of this comparison for WERNER equipped with the 1000P and the PeakFinder is shown in Fig. 5. The PeakFinder measurement was shifted offline by −0.5 mm in depth and scaled by 0.097 in relative dose to reach the best optical agreement over the full measurement range. The shape of both measurements matches well and only small deviations are visible in the high dose gradient regions, which can be attributed to different active volumes of the respective ionization chambers. The high level of agreement after a constant shift in depth is a testament to the precision of the presented motion system.

## 4 DISCUSSION

The vendor‐agnostic water phantom WERNER was successfully validated with proton and carbon beams at MIT and employing the PTW OCTAVIUS 1500XDR as well as the OCTAVIUS 1000P ionization chamber array detectors. Both detectors showed a comparable performance in all investigated scenarios. Therefore, the detector choice purely depends on the intended use case. The PTW 1000P is the detector of choice for areas of interest less than 5.5 × 5.5 cm^2^, due to its higher spatial resolution compared to the PTW 1500XDR at its center. For cases with a larger area of interest, the PTW 1500XDR is the appropriate choice, due to its homogeneous distribution over the full sensitive area.

The measured positional accuracy and reproducibility are comparable to the technical specification of the medical product IBA DigiPhant PT^13^ and were verified via a comparative measurement with the PTW PeakFinder. Compared to an automatic measurement system using array detectors in combination with water‐equivalent plastic slabs,^10^ WERNER does not show any air gap induced build‐up or geometry effects. While PinPoint‐based dosimetry systems are the gold‐standard in absolute dosimetry in water, a full characterization of a complex dose distribution, as needed for the development of for example, 3DRMs, is not feasible due to a significantly lower amount of measurement points per unit time compared to a system such as WERNER. Even though dosimetry approaches using radiographic films or gels offer a high spatial resolution and fast measurement time, the measurement quality and linearity, especially for carbon ions, is highly limited^16^ and not suitable for the intended purpose of the presented system. However, it is important to note that, compared to those systems, a substantial increase in the number of measurement steps to attain a higher measurement resolution will increase the measurement time for a system like WERNER. Therefore, we recommend that WERNER be used with a variable step size, employing larger steps for low dose gradient regions and smaller steps for high dose gradient regions. Promising novel approaches, like GEM‐based systems,^17^ are still in the development phase and not yet suitable for clinical measurements due to strong limitations in active detector area as well as an incomplete understanding of detector response. In contrast, 2D ionization chamber array detectors are well‐understood and routinely used in particle therapy.

WERNER can be fully disassembled for easy transportation to experimental sites and preparations prior to an experiment take around 30 to 60 min, including the full assembly of the system, the filling with demineralized (VE) water and its calibration. It is worth noting that error values obtained during mechanical calibration cannot be directly translated to an absolute measurement error: they are only used as an indicator for the goodness of the mechanical alignment and the irradiation of a monoenergetic scanned field of an appropriate size is strongly advisable as an in‐beam calibration after assembly.

Due to the versatile design of the system and its applicability at a variety of particle accelerators, specific parameters of WERNER, such as detector resolution and measurement speed, are highly dependent on the specific use case and measurement setup. The presented system can potentially work with a multitude of different medical array detectors and is limited mainly by their size, weight, and readout characteristics (time resolved readout mode or external trigger signal are required). By using external signals to control the measurement logic, WERNER can be synchronized to additional measurement equipment, if needed.

## 5 CONCLUSION

A vendor‐agnostic water phantom for 2D ionization chamber array detectors was developed and verified with beam using 150.68 MeV protons and 285.35 MeV/u ^12^C, provided by the medical synchrotron at MIT. The presented system is suitable to be used for the fast characterization of complex dose distributions, as found in many particle therapy related contexts.

## CONFLICT OF INTEREST

The authors report no conflict of interest.
